# Supplementary material for: An open-source framework for large-scale, flexible evaluation of biomedical text mining systems
Source: J Biomed Discov Collab. 2008 Jan 29;3:1. doi: 10.1186/1747-5333-3-1 (PMC2276192; doi:10.1186/1747-5333-3-1)
Supplement: Additional file 1 — Evaluation platform source code. The additional file contains the software evaluation framework discussed in this paper. The most current release can be downloaded from SourceForge.net [1]. See the accompanying README file for instructions on installation and use. Also included in the distribution is a collection of UIMA wrappers for some commonly used BioNLP tools and annotated corpora. [file 1747-5333-3-1-S1.zip › bionlp_uima_repository-1.0.1/docs/api/edu/uchsc/ccp/uima/annotation/syntactic/CCPClauseAnnotation_Type.html]

CCPClauseAnnotation\_Type (Center for Computational Pharmacology Util/UIMA API)


|  |  |  |
| --- | --- | --- |
| **Overview** \| **Package** \| **Class** \| **Tree** \| **Deprecated** \| **Index** \| **Help** | |  |
| **PREV CLASS**   **NEXT CLASS** | **FRAMES**    **NO FRAMES**     **All Classes** |  |
| SUMMARY: NESTED \| FIELD \| CONSTR \| METHOD | DETAIL: FIELD \| CONSTR \| METHOD |  |


---


## edu.uchsc.ccp.uima.annotation.syntactic Class CCPClauseAnnotation\_Type

```
java.lang.Object
  org.apache.uima.jcas.cas.TOP_Type
      org.apache.uima.jcas.cas.AnnotationBase_Type
          org.apache.uima.jcas.tcas.Annotation_Type
              edu.uchsc.ccp.uima.annotation.CCPAnnotation_Type
                  edu.uchsc.ccp.uima.annotation.CCPTextAnnotation_Type
                      edu.uchsc.ccp.uima.annotation.CCPSyntacticAnnotation_Type
                          edu.uchsc.ccp.uima.annotation.syntactic.CCPClauseAnnotation_Type
```

---

``` public class CCPClauseAnnotation_Type extends CCPSyntacticAnnotation_Type ```

This annotation type is used to annotation clauses.
Updated by JCasGen Tue Aug 28 16:44:10 MDT 2007

---

| **Field Summary** | |
| --- | --- |
| `static boolean` | `featOkTst` |
| `static int` | `typeIndexID` |

**Fields inherited from class org.apache.uima.jcas.cas.TOP\_Type**

`casImpl, casType, casTypeCode, instanceOf_Type, jcas, ll_cas, lowLevelArrayBoundChecks, lowLevelTypeChecks, useExistingInstance`


**Constructor Summary**

`CCPClauseAnnotation_Type(org.apache.uima.jcas.JCas jcas,
org.apache.uima.cas.Type casType)`
  
          initialize variables to correspond with Cas Type and Features


| **Method Summary** | |
| --- | --- |
| `String` | `getClauseType(int addr)` |
| `protected  org.apache.uima.cas.impl.FSGenerator` | `getFSGenerator()` |
| `void` | `setClauseType(int addr, String v)` |

**Methods inherited from class edu.uchsc.ccp.uima.annotation.CCPTextAnnotation\_Type**

`getAnnotationID, getAnnotationSets, getAnnotationSets, getAnnotator, getClassMention, getDocumentSectionID, getNumberOfSpans, getSpans, getSpans, setAnnotationID, setAnnotationSets, setAnnotationSets, setAnnotator, setClassMention, setDocumentSectionID, setNumberOfSpans, setSpans, setSpans`

**Methods inherited from class org.apache.uima.jcas.tcas.Annotation\_Type**

`getBegin, getCoveredText, getEnd, setBegin, setEnd`

**Methods inherited from class org.apache.uima.jcas.cas.AnnotationBase\_Type**

`getSofa, getView`

**Methods inherited from class org.apache.uima.jcas.cas.TOP\_Type**

`addToIndexes, checkType, getTypeIndexID, invalidTypeArg, noObjCreate, removeFromIndexes`

**Methods inherited from class java.lang.Object**

`clone, equals, finalize, getClass, hashCode, notify, notifyAll, toString, wait, wait, wait`

**Field Detail**

### typeIndexID

```
public static final int typeIndexID
```

---


### featOkTst

```
public static final boolean featOkTst
```


**Constructor Detail**

### CCPClauseAnnotation\_Type

```
public CCPClauseAnnotation_Type(org.apache.uima.jcas.JCas jcas,
                                org.apache.uima.cas.Type casType)
```

:   initialize variables to correspond with Cas Type and Features


**Method Detail**

### getFSGenerator

```
protected org.apache.uima.cas.impl.FSGenerator getFSGenerator()
```

:   **Overrides:**: `getFSGenerator` in class `CCPSyntacticAnnotation_Type`

---


### getClauseType

```
public String getClauseType(int addr)
```

---


### setClauseType

```
public void setClauseType(int addr,
                          String v)
```


---


|  |  |  |
| --- | --- | --- |
| **Overview** \| **Package** \| **Class** \| **Tree** \| **Deprecated** \| **Index** \| **Help** | |  |
| **PREV CLASS**   **NEXT CLASS** | **FRAMES**    **NO FRAMES**     **All Classes** |  |
| SUMMARY: NESTED \| FIELD \| CONSTR \| METHOD | DETAIL: FIELD \| CONSTR \| METHOD |  |


---
